# Forebrain Cholinergic Signaling Regulates Innate Immune Responses and Inflammation

**DOI:** 10.3389/fimmu.2019.00585

**Published:** 2019-04-02

**Authors:** Kurt R. Lehner, Harold A. Silverman, Meghan E. Addorisio, Ashbeel Roy, Mohammed A. Al-Onaizi, Yaakov Levine, Peder S. Olofsson, Sangeeta S. Chavan, Robert Gros, Neil M. Nathanson, Yousef Al-Abed, Christine N. Metz, Vania F. Prado, Marco A. M. Prado, Kevin J. Tracey, Valentin A. Pavlov

**Affiliations:** ^1^Zucker School of Medicine at Hofstra/Northwell, Hempstead, NY, United States; ^2^Center for Biomedical Science and Bioelectronic Medicine, The Feinstein Institute for Medical Research, Northwell Health, Manhasset, NY, United States; ^3^Schulich School of Medicine and Dentistry, Robarts Research Institute, University of Western Ontario, London, ON, Canada; ^4^Department of Physiology and Pharmacology, Schulich School of Medicine and Dentistry, University of Western Ontario, London, ON, Canada; ^5^Department of Anatomy, Faculty of Medicine, Kuwait University, Kuwait City, Kuwait; ^6^SetPoint Medical Corporation, Valencia, CA, United States; ^7^Department of Medicine, Center for Bioelectronic Medicine, Center for Molecular Medicine, Karolinska Institutet, Karolinska University Hospital, Stockholm, Sweden; ^8^Department of Medicine, Schulich School of Medicine and Dentistry, University of Western Ontario, London, ON, Canada; ^9^Department of Pharmacology, University of Washington, Seattle, WA, United States; ^10^Department of Medicinal Chemistry, Center for Molecular Innovation, The Feinstein Institute for Medical Research, Northwell Health, Manhasset, NY, United States; ^11^Department of Anatomy and Cell Biology, Schulich School of Medicine and Dentistry, University of Western Ontario, London, ON, Canada; ^12^Graduate Program in Neuroscience, Schulich School of Medicine and Dentistry, University of Western Ontario, London, ON, Canada

**Keywords:** forebrain cholinergic, cytokines, inflammation, vagus nerve, endotoxemia, sepsis, neural regulation

## Abstract

The brain regulates physiological functions integral to survival. However, the insight into brain neuronal regulation of peripheral immune function and the neuromediator systems and pathways involved remains limited. Here, utilizing selective genetic and pharmacological approaches, we studied the role of forebrain cholinergic signaling in the regulation of peripheral immune function and inflammation. Forebrain-selective genetic ablation of acetylcholine release and vagotomy abolished the suppression of serum TNF by the centrally-acting cholinergic drug galantamine in murine endotoxemia. Selective stimulation of acetylcholine action on the M1 muscarinic acetylcholine receptor (M1 mAChR) by central administration of the positive allosteric modulator benzyl quinolone carboxylic acid (BQCA) suppressed serum TNF (TNFα) levels in murine endotoxemia. This effect was recapitulated by peripheral administration of the compound. BQCA also improved survival in murine endotoxemia and these effects were abolished in M1 mAChR knockout (KO) mice. Selective optogenetic stimulation of basal forebrain cholinergic neurons innervating brain regions with abundant M1 mAChR localization reduced serum TNF in endotoxemic mice. These findings reveal that forebrain cholinergic neurons regulate innate immune responses and inflammation, suggesting the possibility that in diseases associated with cholinergic dysfunction, including Alzheimer's disease this anti-inflammatory regulation can be impaired. These results also suggest novel anti-inflammatory approaches based on targeting forebrain cholinergic signaling in sepsis and other disorders characterized by immune dysregulation.

## Introduction

The nervous system regulates and coordinates physiological functions and defense mechanisms. A major defense mechanism against pathogen invasion and tissue injury is provided by the innate immune system through inflammation (1). However, dysregulated immune responses and aberrant inflammation are implicated in the etiology of sepsis, inflammatory bowel disease, and many other life-threatening and debilitating disorders (1–6). While accumulating evidence reveals that the nervous system and specifically the vagus nerve regulate immune function and inflammation, the role of brain pathways in this context remains poorly understood (2, 7, 8). The brain regulation of peripheral inflammation and the mediating role of the vagus nerve have been indicated in studies with the experimental anti-inflammatory compound CNI-1493 (Semapimod). Administration of this molecule in the brain suppresses serum TNF (TNF-α) in murine endotoxemia and this effect is abrogated by surgical transection of the vagus nerve (vagotomy) (9). CNI-1493 binds to muscarinic acetylcholine receptors (mAChRs) (10) and administration of compounds that mimic the action of acetylcholine on mAChRs in the brain also suppresses circulating TNF and other pro-inflammatory cytokines (10–12). In addition, galantamine, an acetylcholinesterase inhibitor, which increases acetylcholine levels suppresses peripheral pro-inflammatory cytokine levels acting through a brain mAChR-mediated mechanism (13–15). These cholinergic effects in the brain are linked with activation of the vagus nerve-based inflammatory reflex (11, 13, 14, 16, 17), a physiological immunoregulatory circuit (18, 19) with recently demonstrated utility in treating human inflammatory diseases (20, 21). These studies have implicated brain cholinergic signaling in the regulation of pro-inflammatory cytokine release and inflammation. The cholinergic system in the brain has a diverse topographic neuronal organization and projection patterns (22, 23), and specific insight into the role of cholinergic pathways and receptors in the brain in peripheral immunoregulation is presently lacking.

A major collection of cholinergic neurons is localized in the basal forebrain (22, 24). These neurons project to forebrain regions with abundant expression of M1 mAChRs, including neocortical areas and the hippocampus (23), and regulate neuroplasticity, cognition, and other processes (22, 23, 25). Here, utilizing mice with selective genetic forebrain ablation of acetylcholine release, positive allosteric M1 mAChR modulation, and optogenetic stimulation of basal forebrain cholinergic neurons, we indicate a role for forebrain cholinergic signaling via M1 mAChRs in the physiological regulation of inflammation.

## Results

### Acetylcholine in Forebrain Mediates Cholinergic Suppression of Peripheral Pro-inflammatory Cytokine Release via a Vagus Nerve-Dependent Signaling

**Figure 1:**
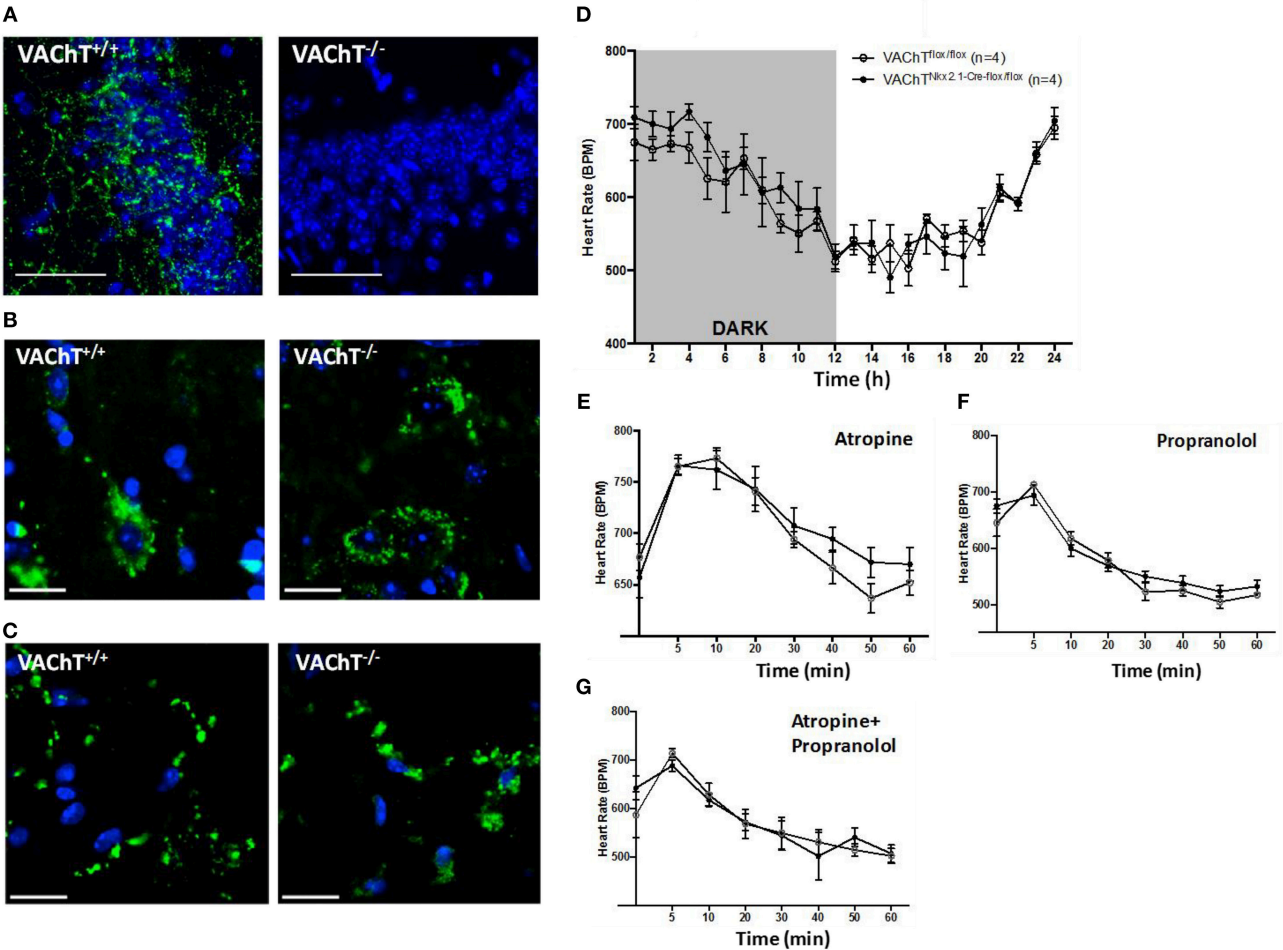
Morphological and functional evaluation of forebrain VAChT KO mice. **(A)** VAChT protein immunostaining (in green) shown in a hippocampal region of VAChT^+/+^ mice is not detected in VAChT^−/−^ mice (scale bar = 100 μm). **(B)** VAChT protein immunostaining (in green) in spinal cord of VAChT^+/+^ and VAChT^−/−^ mice (scale bar = 20 μm). **(C)** VAChT protein immunostaining (in green) in the heart right atrium of VAChT^+/+^ and VAChT^−/−^ mice (scale bar = 20 μm). **(D)** Freely moving VAChT^flox/flox^ (VAChT^+/+^) mice and VAChT ^Nkx2.1−Cre−flox/flox^ (VAChT^−/−^) mice exhibit no differences in heart rate during 24 h recording using radio-frequency telemeters. Freely moving VAChT^+/+^ (open circles) and VAChT^−/−^ mice (black circles) exhibit no difference in heart rate responses to atropine **(E)**, propranolol **(F)**, or atropine and propranolol **(G)** i.p. administration (*n* = 4 mice/genotype).

To investigate the role of forebrain neuronal acetylcholine in regulating peripheral inflammation, we utilized mice with selective forebrain deprivation of acetylcholine release and galantamine, a centrally-acting cholinergic drug (an acetylcholinesterase inhibitor) with anti-inflammatory properties (13, 14). A major molecular determinant of acetylcholine release is the vesicular acetylcholine transporter (VAChT), which loads acetylcholine in vesicles prior to its release in the synaptic cleft (26, 27). We used mice with Cre-*lox*P-based forebrain VAChT ablation, a genetic manipulation that provides a non-invasive means of selective elimination of forebrain acetylcholine release and cholinergic activity without loss of neurons (27). This is important as cholinergic neurons can also secrete GABA with ACh and targeting VAChT allows for selective manipulation of ACh release (28–31). As shown in Figure 1A, no VAChT immunoreactivity in the forebrain (hippocampus, CA3 area shown) was detected in these VAChT^Nkx2.1−Cre−flox/flox^ (VAChT^−/−)^ mice as compared with VAChT^flox/flox^ (VAChT^+/+^) control mice. This observation was consistent with the previously reported lack of VAChT immunoreactivity in several forebrain areas of VAChT^−/−^ mice and no significant alterations in VAChT protein expression in brainstem regions as a result of genetic deletion (27). The selective forebrain VAChT ablation was not associated with differences in VAChT protein levels (immunofluorescence) in the spinal cord and peripheral neuronal varicosities in the heart (Figures 1B,C). These observations indicated that VAChT ablation was limited to the basal forebrain cholinergic system. Furthermore, no differences were observed between VAChT^−/−^ and VAChT^+/+^ mice on 24 h heart rate recording (Figure 1D). There were no differences between the two groups of mice in heart rate responses to atropine (mAChR blocker) and propranolol (beta adrenergic receptor blocker) i.p. administrations (Figures 1E–G). In addition, sensitivity to post-handling stress was similar between genotypes, because heart rate responses to saline administration did not differ between the two groups of mice (Figure S1).

**Figure 2:**
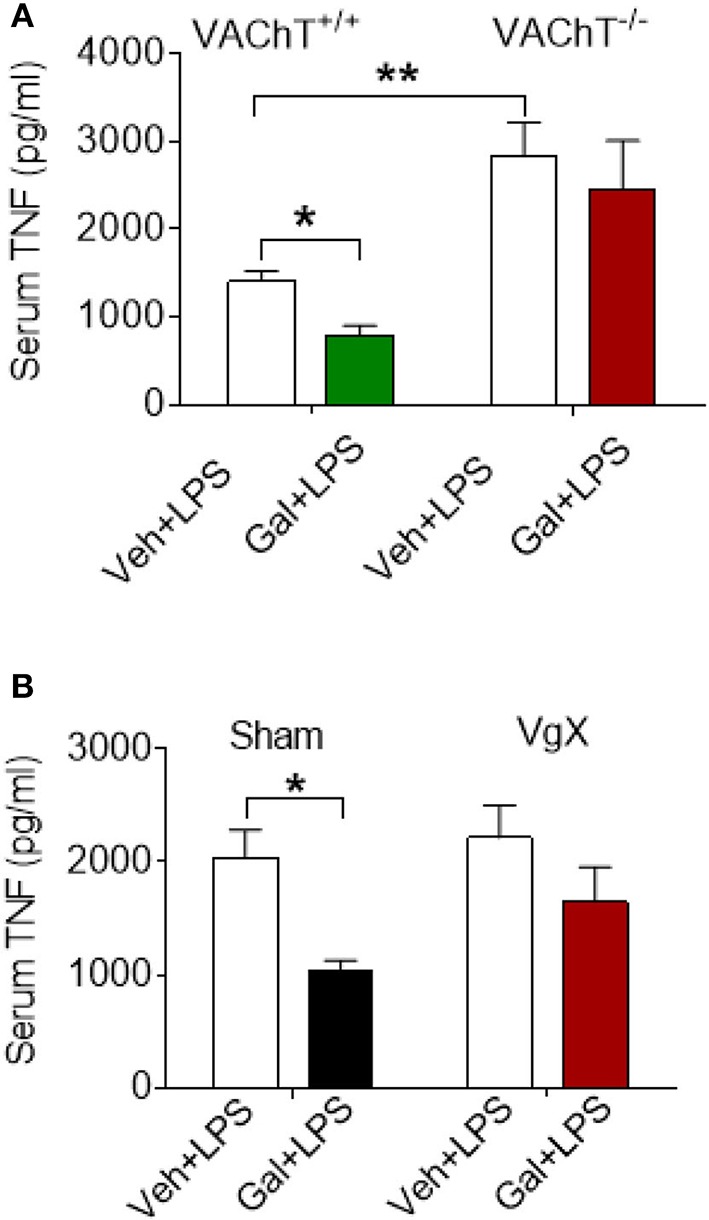
Selective forebrain cholinergic deficit and vagotomy alter cholinergic suppression of peripheral pro-inflammatory cytokine levels. **(A)** Galantamine (Gal, i.p.) as compared to vehicle (Veh), suppresses serum TNF in VAChT^+/+^ control mice, but not in VAChT^−/−^ mice during endotoxemia, and serum TNF in vehicle injected VAChT^−/−^ mice are higher as compared to VAChT^+/+^ control mice (^*^P = 0.017, ^**^P = 0.007, two-way ANOVA, Sidak's multiple comparisons test), *n* = 6–7 per group. **(B)** Vagotomy (VgX) abolishes the suppression of serum TNF in endotoxemic mice (^*^P = 0.019, two-way ANOVA, Sidak's multiple comparisons test), *n* = 6–8 per group. See Methods for details.

The effects of acetylcholine in the brain can be modulated (enhanced) through inhibiting its degradation using centrally-acting acetylcholinesterase inhibitors, including galantamine. Administration (i.p.) of galantamine in VAChT^+/+^ mice prior to endotoxin significantly reduced serum TNF levels as compared to vehicle administration (*P* = 0.017) (Figure 2A). However, galantamine failed to significantly alter serum TNF levels in VAChT^−/−^ mice (Figure 2A). In addition, serum TNF levels in VAChT^−/−^ mice were significantly higher than in VAChT^+/+^ mice during endotoxemia (*P* = 0.009) (Figure 2A), suggesting that physiological cholinergic transmission in the forebrain regulates peripheral innate immune responses. Galantamine was previously shown to stimulate vagus nerve activity (17, 32). We next examined the role of the vagus nerve in mediating galantamine forebrain-triggered anti-inflammatory effect in endotoxemic C57BL/6 mice. Galantamine administration significantly decreased serum TNF levels as compared with vehicle during endotoxemia in sham-operated (control) mice (*P* = 0.019) (Figure 2B). This effect was markedly diminished in mice with cervical unilateral vagotomy (Figure 2B), thus indicating a role of the efferent vagus nerve. Collectively, these data indicate that acetylcholine derived from cholinergic neurons in forebrain plays a role in mediating the suppressive effect of galantamine on peripheral TNF levels in a vagus nerve-dependent manner during murine endotoxemia.

### Stimulation of Acetylcholine Action on the M1 mAChR by Allosteric Modulation Suppresses Lethal Peripheral Inflammation

**Figure 3:**
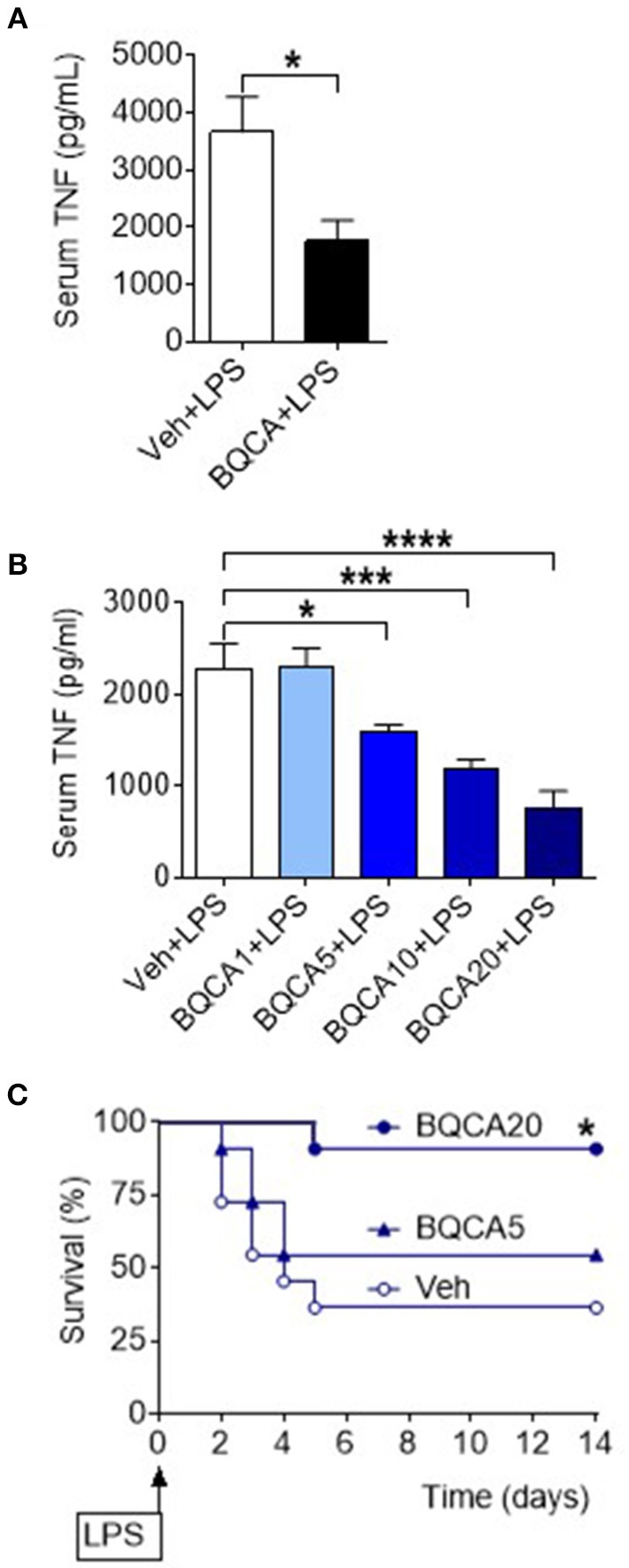
Activation of acetylcholine action on the M1 mAChR using a positive allosteric modulator (BQCA) suppresses serum TNF levels and improves survival in endotoxemia. **(A)** BQCA (5 μ/kg, i.c.v.) suppresses serum TNF (^*^P = 0.018, unpaired two-tailed Student's *t* test), *n* = 6–7 per group. **(B)** Peripheral i.p. administration of BQCA 1–20 (1–20 mg/kg) in endotoxemic mice suppresses serum TNF levels (^*^P = 0.027, ^***^P = 0.0005, ^****^P < 0.0001, one-way ANOVA, Dunnett's multiple comparisons test), *n* = 7–8 per group. **(C)** Peripheral i.p. administration of BQCA 20 (20 mg/kg) in endotoxemic mice improves survival in endotoxemia (^*^P = 0.035, Log-rank test), *n* = 11–12 per group.

Basal forebrain cholinergic neurons project to regions with high expression of M1 mAChR, including several neocortical areas and the hippocampus. Acetylcholine released from these neurons stimulates the postsynaptically located M1 mAChR that processes cholinergic transmission (33). Positive allosteric modulation of the M1 mAChR is a selective approach of increasing endogenous acetylcholine activity at the receptor (34, 35). To study the effect of acetylcholine on brain M1 mAChR in the regulation of peripheral inflammation, we used benzyl quinolone carboxylic acid (BQCA), a positive allosteric modulator of M1 mAChR (34, 35). BQCA has previously been shown to selectively increase (up to 129-fold) the functional affinity of endogenous acetylcholine for M1 mAChR (35). Intracerebroventricular (i.c.v.) injection of BQCA (5 μg/kg) resulted in significant suppression of serum TNF as compared with vehicle administration following endotoxin challenge (*P* = 0.018) (Figure 3A). This observation indicated that selective activation of acetylcholine action on the M1 mAChR in the brain suppresses peripheral inflammation in murine endotoxemia. To facilitate subsequent studies with BQCA, we examined whether peripheral administration of BQCA, which is known to cross the blood brain barrier (34, 35), recapitulates anti-inflammatory effects. Intraperitoneal (i.p.) treatment of mice with BQCA (1–20 mg/kg) resulted in a dose-dependent decrease in serum TNF following endotoxin as compared to vehicle injection (*P* < 0.001, when 20 mg/kg BQCA was used) (Figure 3B). I.p. administration of BQCA also dose-dependently improved the survival rate in this lethal murine endotoxemia model as compared to vehicle administration (*P* = 0.035 when 20 mg/kg BQCA was used) (Figure 3C).

**Figure 4:**
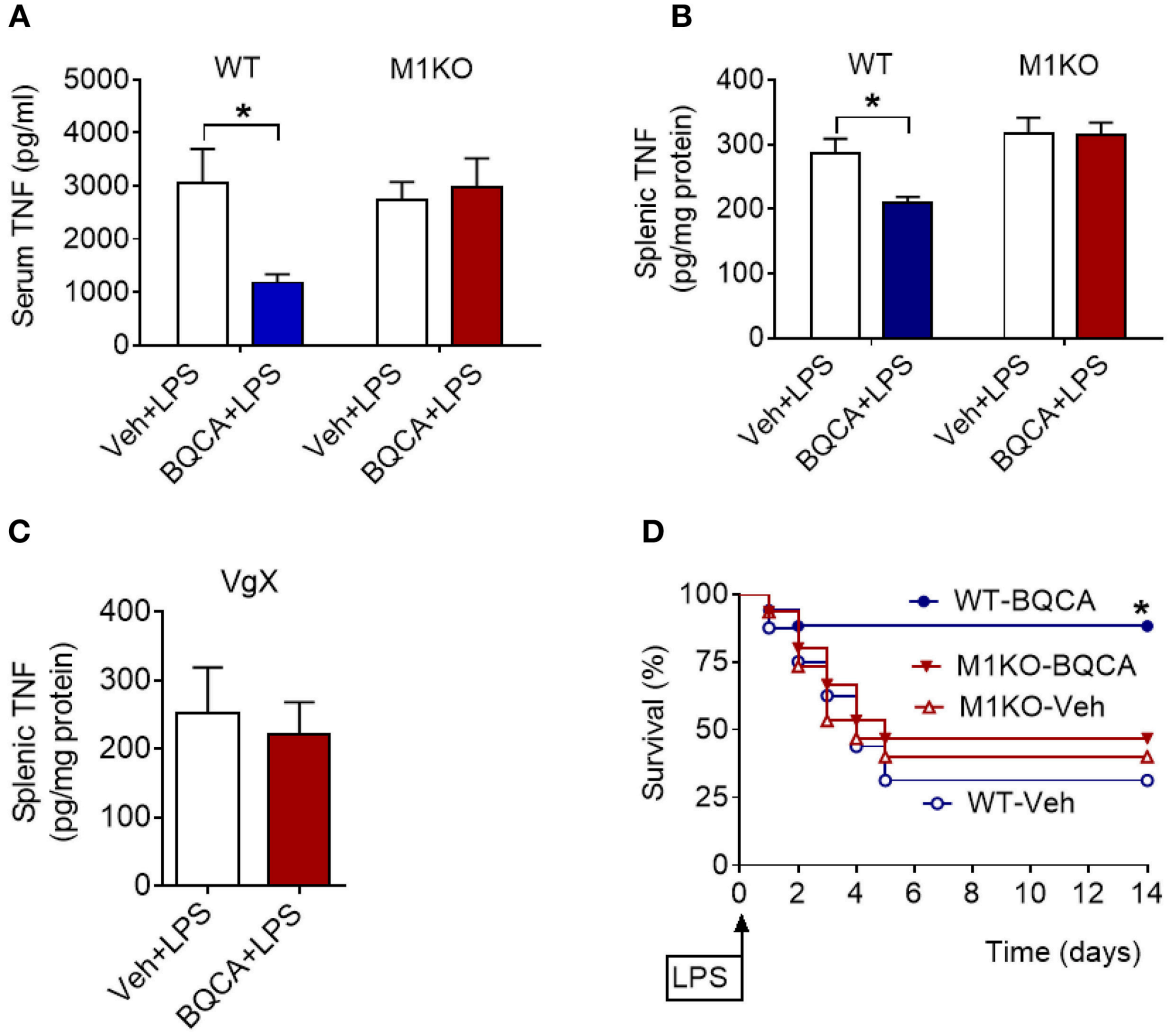
Anti-inflammatory effects of BQCA in endotoxemia are mediated by M1 mAChRs. **(A)** Peripheral (i.p.) administration of BQCA (20 mg/kg, i.p.) suppresses serum TNF in wild type (WT) mice and does not alter serum TNF in M1 mAChR KO mice during endotoxemia (^*^P = 0.01, two-way ANOVA, Sidak's multiple comparisons test), *n* = 8–10. **(B)** Peripheral (i.p.) administration of BQCA (20 mg/kg, i.p.) suppresses splenic TNF in WT mice and does not alter splenic TNF in M1 mAChR KO mice (^*^P = 0.017, two-way ANOVA, Sidak's multiple comparisons test), *n* = 7–10. **(C)** Vagotomy (VgX) abolishes the effect of BQCA on splenic TNF during endotoxemia (unpaired two-tailed Student's *t* test), *n* = 7, 8. **(D)** Peripheral (i.p.) administration of BQCA (20 mg/kg, i.p.) improves survival in endotoxemic WT mice and does not alter the survival rate in M1 mAChR KO mice during endotoxemia (^*^P = 0.028, Log-rank test), *n* = 15–18 per group. See Methods for details.

BQCA treatment (20 mg/kg, i.p.) did not significantly alter serum TNF in M1 mAChR KO mice (as compared to vehicle treatment), while in wild type (WT) mice this drug effect was significant (*P* = 0.028) (Figure 4A). Previous studies have shown that activation of brain mAChR-mediated cholinergic signaling results in suppression of TNF in spleen, a major source of pro-inflammatory cytokines and an organ target of the vagus-nerve-based inflammatory reflex (36–38). BQCA (i.p.) administration 1 h prior to endotoxin significantly suppressed splenic TNF in WT mice (*P* = 0.047) and did not alter splenic TNF in M1 mAChR KO mice (Figure 4B). BQCA also failed to significantly alter splenic TNF in endotoxemic WT mice with unilateral cervical vagotomy (Figure 4C). In addition, BQCA (20 mg/kg, i.p.) injected 1 h prior to endotoxin was sufficient to significantly improve survival in WT mice (*P* = 0.028), but failed to significantly alter survival in endotoxemic M1 mAChR KO mice (Figure 4D). Together these results show that enhancement of acetylcholine activity on the M1 mAChR is sufficient to suppress inflammation in murine endotoxemia and that the efferent vagus nerve is necessary for this anti-inflammatory effect.

### Selective Optogenetic Stimulation of Basal Forebrain Medial Septum Cholinergic Neurons Suppresses Serum TNF Levels

**Figure 5:**
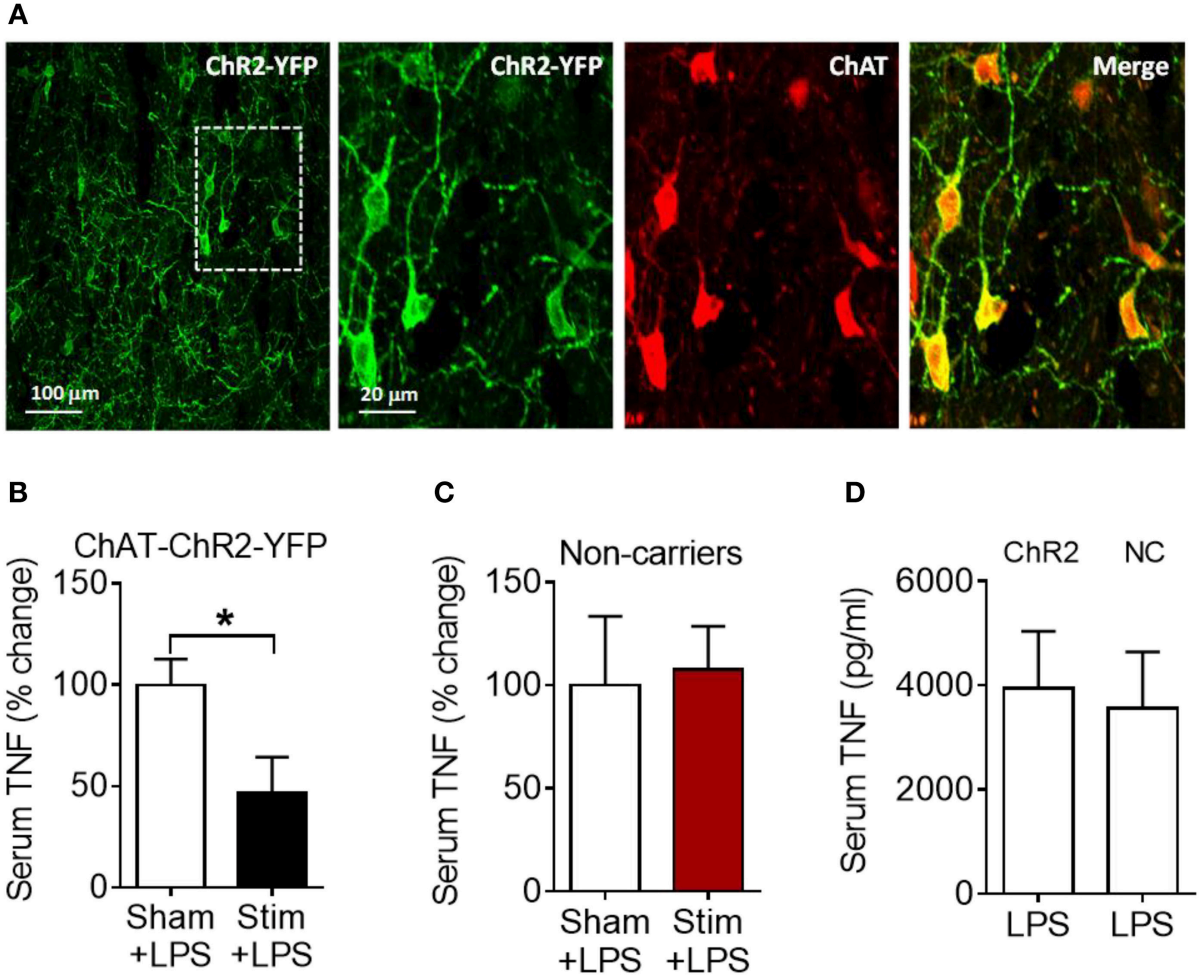
Optogenetic stimulation of basal forebrain cholinergic neurons in the medial septum suppresses serum TNF in endotoxemic mice. **(A)** Immunostaining of medial septum neurons in a brain section of a ChAT-ChR2-EYFP mouse: EYFP immunostaining (first and second panel); ChAT immunostaining of the same area (third panel); and double immunostaining (fourth panel). **(B)** Optogenetic stimulation suppresses serum TNF during endotoxemia (^*^P = 0.039, unpaired two-tailed Student's *t* test), *n* = 5–6 per group. **(C)** Laser light exposure of medial septum neurons in control (non-carrier) mice does not significantly alter serum TNF levels during endotoxemia (unpaired two-tailed Student's *t* test), *n* = 6–7 per group. **(D)** LPS (i.p.) administration to ChAT-ChR2-EYFP (ChR2) and control (non-carrier, NC) mice does not result in statistically different serum TNF levels, (unpaired two-tailed Student's *t* test), *n* = 9 per group. See Methods for details.

We next examined whether the anti-inflammatory effect of acetylcholine acting on M1 mAChR (achieved by allosteric modulation with BQCA) could be replicated by direct stimulation of basal forebrain cholinergic neurons *in vivo*. The medial septum (medial septal nucleus) is a major nucleus in the basal forebrain cholinergic system (22, 23). The medial septum also plays an important role as a relay of afferent vagus nerve signaling in the forebrain as recently demonstrated (39). Accordingly, we stimulated basal forebrain medial septum cholinergic neurons using a selective optogenetic approach in transgenic mice. These mice express channelrhodopsin-2 coupled to a yellow fluorescent protein (ChR2-YFP) under the control of the choline acetyltransferase (ChAT) promoter. Immunofluorescent staining of brain slices confirmed the neuronal colocalization of ChAT and ChR2-YFP in the medial septum (Figure 5A) and the abundant expression of ChR2-YFP in the medial septum and the adjacent vertical limb of the diagonal band of Broca (Figure S2). Photoactivation of medial septum neurons by laser light (473 nm) significantly suppressed serum TNF levels compared with sham stimulation (*P* = 0.039) (Figure 5B) in mice with confirmed (by microscopic histochemical evaluation) location of the fiber tip in the medial septum (Figure S3; Supplementary Methods). Laser light exposure of medial septum neurons in C57BL/6 mice not expressing ChR2-YFP on cholinergic neurons (non-carriers) was performed to control for possible confounding effects of heat and other non-thermal effects of light. This manipulation failed to alter serum TNF levels (Figure 5C) thus confirming the specific cholinergic nature of the mechanism. As ChAT-ChR2-YFP mice have been shown to overexpress VAChT (40, 41) and present increased cholinergic tone, LPS was administered to ChAT-ChR2-YFP and non-carrier mice not subjected to anesthesia and surgical manipulation. No differences in serum TNF levels upon LPS administration were observed between the two groups of mice (Figure 5D), indicating that the genetic modification by itself did not alter the inflammatory response. These results show that selective activation of basal forebrain medial septum cholinergic neurons decreases peripheral inflammation in endotoxemia.

## Discussion

Here, we show a role for forebrain cholinergic signaling and the M1 mAChR in the brain neuronal regulation of inflammation through vagus nerve-mediated signaling.

Genetic ablation of VAChT, which eliminates synaptic acetylcholine release from basal forebrain cholinergic neurons (27), abolished the anti-inflammatory effect of the centrally-acting acetylcholinesterase inhibitor galantamine. These findings point to forebrain acetylcholine as a major mediator of galantamine anti-inflammatory effects in endotoxemia. The selective forebrain cholinergic deficit (in VAChT^−/−^ mice) also resulted in increased circulatory TNF levels in endotoxemic mice, thus suggesting a tonic anti-inflammatory role of acetylcholine in forebrain.

Forebrain areas, including the cortex (neocortex) and the hippocampus have abundant (predominantly post-synaptic) localizations of the M1 mAChR, which plays a major role in processing the effects of acetylcholine released from basal forebrain cholinergic neurons (22). Positive allosteric modulation of the M1 mAChR by BQCA is a selective approach of increasing the activity of the endogenous acetylcholine on the receptor (34, 35). The anti-inflammatory effects of the centrally-acting BQCA demonstrated here indicate a role of acetylcholine acting on the M1 mAChR in controlling peripheral inflammatory responses. Unilateral vagotomy attenuated the anti-inflammatory effects of galantamine and BQCA, thus indicating a brain-to periphery mediating role for the vagus nerve. The vagus nerve is an important neuroanatomical component of the inflammatory reflex (18) in which sensory and motor vagus nerve signaling regulates cytokine production by immune cells in the spleen, and alleviates inflammation (2, 19). Importantly, recent studies demonstrated that afferent vagus nerve signaling reaches the forebrain (hippocampus and cortex), and identified basal forebrain cholinergic nuclei, including the medial septum as major relay components (39, 42, 43). Together with these findings, our observations suggest a role for the medial septum in a forebrain regulatory hub of the inflammatory reflex. This regulation possibly involves other brain regions through multisynaptic pathways with brainstem nuclei providing peripheral vagus nerve projections. These brain networks remain to be further elucidated.

In addition to positive allosteric modulation of acetylcholine action on M1 mAChR, suppression of serum TNF levels during endotoxemia was also achieved by selective optogenetic stimulation of a subset of basal forebrain cholinergic neurons in the medial septum. Although optogenetic stimulation was performed in animals under isoflurane anesthesia and there is some evidence that relatively high doses of isoflurane can increase blood-brain barrier permeability (44), the proper sham stimulation pointed to the specificity of the effect. Medial septum cholinergic neurons innervate the hippocampus and the parahippocampal region, which are interconnected with several neocortical and subcortical areas (23, 24, 39). Multisynaptic connections between the hippocampus and the hypothalamus have also been described. Although we stereotactically targeted optogenetic stimulation of the medial septum, a spread of stimulation to adjacent cholinergic neurons in the diagonal band of Broca cannot be excluded. Cholinergic neurons in the diagonal band of Broca project to cortical areas and the amygdala (22–24). These neural networks involving cholinergic circuits suggest that multiple points of regulation can be further explored in studying the brain control of peripheral inflammation. Some of these networks have been previously associated with the regulation of autonomic responses, including modulation of vagus nerve activity (2). These previous observations are consistent with our findings that signaling through the vagus nerve mediates brain cholinergic modulation of peripheral inflammation. Our findings have clinical relevance. Galantamine is in clinical use for alleviation of cognitive deterioration in patients with Alzheimer's disease. We have also recently shown that treatment with galantamine alleviates inflammation and insulin resistance in patients with the metabolic syndrome (45). BQCA and other centrally-acting positive allosteric modulators of the M1 mAChR have also been preclinically developed in the search of efficient treatments of Alzheimer's disease and other neurological conditions (34, 35). Our results suggest considering these two types of therapeutics as anti-inflammatory agents. While optogenetics provide a very selective approach for spatiotemporal neuronal control in studying neural circuitries (46), this technology currently has limited therapeutic implications (47–49). However, medial septum deep brain (electrical) stimulation has been successfully used for improving spatial working memory and cognition in experimental settings of traumatic brain injury (50, 51). Future insights into brain networks triggered by medial septum neuronal stimulation and possibly other regions may further inform implications of deep brain stimulation and non-invasive approaches, including transcranial magnetic stimulation and transcranial direct current stimulation (52–54) in therapeutic anti-inflammatory strategies.

Basal forebrain cholinergic signaling has a documented role in attention, learning and memory, and degeneration of basal forebrain cholinergic neurons, which results in diminished release of acetylcholine in the forebrain, is one of the hallmarks of Alzheimer's disease (23). Intriguingly, increased peripheral TNF and other pro-inflammatory cytokine levels have been found in Alzheimer's disease (55), and peripheral inflammation has been linked to exacerbation of brain pathogenesis and neurodegeneration, affecting cholinergic neurons (56, 57). This may be of importance given that cholinergic neurotransmission is dysfunctional in different types of dementia (58), suggesting a potential mechanism by which patients with Alzheimer's disease and other types of dementia may be more susceptible to distinct types of infection (59).

Peripheral inflammation in sepsis, liver disease, and other inflammatory conditions also is linked with brain inflammation (2, 60) and deterioration in brain function and delirium within the scope of characteristic encephalopathies (2, 61–64). Dysregulation in cholinergic signaling has a documented role in brain derangements, including delirium and encephalopathies in sepsis and other inflammatory conditions (60, 65–67). Together with these previously published findings, our observations suggest a bidirectional relationship between brain cholinergic signaling and peripheral inflammation. In this context targeted pharmacological or device-generated modulation of forebrain cholinergic signaling may have broader therapeutic implications.

## Materials and Methods

### Animals

Male mice were used in all experiments. BALB/c mice (24–28 g) were purchased from Taconic. Mice with genetic deletion of VAChT in the forebrain (VAChT^–/–^ mice) were generated as previously described (27). Briefly, VAChT^flox/flox^ mouse line (crossed for five generations with C57BL/6J) was crossed with the Nkx2.1-Cre mouse line (C57BL/6J-Tg(Nkx2-1-cre)2Sand/J) (Jackson Laboratory, stock No: 008661). Control VAChT^+/+^ mice were VAChT^flox/flox^ littermates. ChAT-ChR2-YFP BAC transgenic mice (Jackson Laboratory, stock No: 014546) (68) and non-carrier wild type mice (on the C57BL/6 background) were used in experiments with laser light exposure of the medial septum neurons. M1 mAChR *(Chrm1*^−/−^) KO mice (69) were generated on the C57Bl/6 background as previously described (70). M1 mAChR KO and wild type (WT) littermates were received from Charles River. Animals were allowed to acclimate for at least 2 weeks prior to the corresponding experiment. All animals were housed in standard conditions (a 12 h light–dark cycle) with access to regular chow and water.

### Chemicals

Galantamine (Galantamine hydrobromide) (purity ≥95% by HPLC) was purchased from Calbiochem. Galantamine working solutions were prepared in sterile saline. Benzyl quinolone carboxylic acid (BQCA) (purity: ≥97% (HPLC) was purchased from Enzo Life Sciences. BQCA working solutions were prepared in betacyclodextrin and contained 5% betacyclodextrin in sterile saline (35). Atropine (purity: ≥99% (TLC) and propranolol (purity: ≥98% (TLC) were purchased from Sigma and working solutions prepared in sterile saline.

### Endotoxemia and Drug Treatment

Endotoxemia in mice was induced by administering LPS (endotoxin, Sigma L4130 O111:B4 for consistency with our previous studies (13, 36, 71, 72) in doses as indicated, injected i.p.) Groups of animals were treated i.p. with galantamine (3 mg/kg) or different doses of BQCA or vehicle (sterile saline as a vehicle in galantamine treatments or 5% betacyclodextrin containing sterile saline as a vehicle in BQCA treatments) 1 h prior to endotoxin administration. Vehicle or BQCA (5 μg/kg) [based on the information available about the effect of the compound on M1 mAChR (34, 35)] was also administered i.c.v. 1 h prior to endotoxin (8 mg/kg, i.p.). In experiments with optogenetic stimulation LPS was injected i.p. 1 h after the end of stimulation or sham stimulation. Animals were euthanized by CO_2_ asphyxiation 1.5 h after endotoxin administration, and blood was collected via cardiac puncture (from the right ventricle) for cytokine (TNF) determination. In other sets of survival experiments, groups of mice were treated with BQCA or vehicle (i.p.) 1 h prior to endotoxin (8 mg/kg, i.p.) injection. Mice were monitored for survival twice daily for the first 5 days, and then daily for the remainder of the 14 day experiments.

### Vagotomy

To avoid potential high lethality associated with bilateral cervical vagotomy, a unilateral cervical vagotomy was performed. Mice were anesthetized by isoflurane inhalation and the right cervical vagus nerve was exposed, ligated with a 4–0 silk suture, and divided. In sham-operated animals, the cervical vagus nerve was visualized, but was neither isolated from the surrounding tissues nor transected. All animals were permitted to recover for 7 days following the surgical procedure and before their inclusion in endotoxemia experiments.

### Brain Surgical Manipulations and Optogenetic Stimulation

In experiments with optogenetic stimulation, mice were anesthetized with isoflurane and placed on a stereotactic frame (Kopf Instruments). Body temperature was maintained between 36.5 and 37°C using a feedback controlled rectal thermometer and heating pad. Using aseptic technique, the scalp was incised to expose the skull and the overlying connective tissue was removed. A small (~500 μm) craniotomy was performed on the desired locations for fiber insertion. Mice expressing light-activated cation ChR2 tagged with a fluorescent protein (ChR2-YFP) under the control of the choline acetyltransferase (ChAT) promoter were used in optogenetic experiments. The optic fiber was inserted slowly over 3 min, targeting the dorsal cholinergic neurons in the medial septum at a location 0.8 mm anterior to Bregma and 0.7 mm lateral to a depth of 3.5 mm at a 10 degree angle below dura, which was opened with a 27 gauge needle. Laser light was delivered via a 200 μm diameter fiber (Thorlabs) inserted into the craniotomy. For ChR2 activation, a 473 nm laser (Optoengine, LLC) was used at a power of 10 mW at the tip. The laser was controlled by a waveform generator (Agilent). Stimulation was performed by using square pulses at 20 Hz for 10 min. Sham stimulation was carried out the same way with the exception that following optic fiber insertion, no laser stimulation was performed. Following optogenetic stimulation or sham stimulation, the fiber was removed over 3 min, the craniotomy was covered using paraffin wax, and the animal recovered for 1 h on a heating pad at 37°C prior to LPS injection. Laser light exposure of medial septum neurons or sham laser light exposure was performed following the same protocol in non-carrier mice.

### Brain Surgical Manipulations and I.C.V. Drug Administration

Craniotomies were performed as described above at a location 0.6 mm posterior and 1.2 mm lateral to Bregma targeting the right lateral ventricle. A 10 μL Hamilton microsyringe in a microinjector pump (UMP3-1, World Precision Instruments) was lowered into the right lateral ventricle over 3 min to a depth of 2.1 mm. Following 5 min of equilibration, BQCA (5 ug/kg) or vehicle was delivered over 3 min. Following injection, the syringe remained in place for 5 min to prevent backflow. Then the needle was slowly removed over 3 min. The craniotomy was covered using paraffin wax and the animal recovered for 1 h on a heating pad at 37° C prior to LPS injection.

### Immunohistochemistry and Immunofluorescent Microscopy

Expression of ChR2-EYFP in cholinergic neurons of the medial septum was confirmed with immunofluorescent staining for choline acetyltransferase (ChAT) and ChR2-YFP. Mice underwent intracardiac perfusion with 1 × PBS followed by 4% paraformaldehyde. Brains were harvested and cryoprotected with subsequent incubations of 15% and 30% sucrose. They were then stored in O.C.T. at −20°C. 20 μm sections were collected on gelatin subbed slides (SouthernBiotech) using a cryostat (Leica Microsystems). Slides were rinsed in 1 × PBS followed by washing with 0.25% Triton X-100/1 × PBS (PBT). Blocking was performed with a solution of 10% MeOH, 0.1% bovine serum albumin (BSA), 3% normal donkey serum (NDS), and 0.05% hydrogen peroxide. Slides were then washed in PBT. Cholinergic neurons were visualized using goat anti-ChAT (Millipore, AB144 1:200, dilution) as a primary antibody and specific ChR2-EYFP expression was visualized using rabbit anti-GFP Alexa Fluor 488 (ThermoFisher Scientific, A21311, 1:400 dilution) in a solution of PBT containing 1% NDS. Donkey anti-goat Alexafluor 555 (Thermo Fisher Scientific, A21432, 1:200 dilution) in a solution of 1% NDS/PBT was used to visualize Goat anti-ChAT, and slides were mounted and coverslipped with DAPI-Fluoromount G (Southern BioTech). Images were taken using an Olympus FluoView FV300 Confocal Laser Scanning Microscope.

In experiments with VAChT^−/−^ and VAChT^+/+^ animals, mice were anesthetized with ketamine (100 mg/kg) and xylazine (25 mg/kg) in 0.9% sodium chloride, and then sacrificed by trans-cardial perfusion. Brains were harvested and placed in 4% paraformaldehyde in 1 × PBS overnight at 4°C. The brains were isolated and 40 μm sections of the tissue were obtained using a vibratome. Brain sections were incubated in a blocking solution of 1 × PBS/0.4% Triton X-100 containing 0.1% glycine (wt/vol), 0.1% lysine (wt/vol), 1% BSA (wt/vol), and 1% normal donkey serum (wt/vol). Sections were incubated with anti-VAChT primary antibody (catalog no. 139103; Synaptic Systems) overnight. Sections were then incubated with the Alexa Fluor 488 anti-rabbit secondary antibody (1:1,000; Life Technologies) for 1 h. The nuclei were labeled with Hoechst. Images were acquired using the Zeiss LSM 510 Meta confocal system as previously described (27).

### Serum Isolation and Cytokine Determination

The blood was allowed to clot for 80 min following collection. It was then centrifuged at 5,000 rpm (1,500 × g) for 10 min, and the supernatant (serum) was collected and stored at −20°C until analysis. Serum TNF was quantified by using ELISA per the manufacturer's instructions (eBioscience).

### Electrocardiography (ECG)

Electrocardiograms were recorded using radiotelemeters. The radio frequency transmitters were implanted subcutaneously under anesthesia and ECG recordings were initiated following a minimum recovery period of 7 days post-implantation. Heart rate was continuously measured in awake, freely moving mice over 24 h to obtain baseline recordings. To determine the effect of autonomic blockade, heart rate was recorded for 60 min following administration of atropine (1 mg/kg, i.p.), propranolol (1 mg/kg, i.p.), or atropine + propranolol. All data were collected using the Dataquest A.R.T. software (Transoma Medical). Experiments were performed as previously described (73, 74).

### Statistical Analysis

GraphPad Prism 6.0 software was used for all statistical analysis. Values are presented as mean ± SEM. One-way or two-way ANOVA, followed by appropriate *post-hoc* tests for multiple comparisons, and a two-tailed two-sample equal variance Student's *t*-test were performed to determine statistical significance. The statistical significance of differences between groups of animals in survival experiments was analyzed by Log-rank test. *P* values equal to or below 0.05 were considered significant.

## Data Availability

All datasets generated for this study are included in the manuscript and/or the Supplementary Files.

## Ethics Statement

All animal experiments were performed in accordance with the National Institutes of Health Guidelines under protocols approved by the Institutional Animal Care and Use Committee and the Institutional Biosafety Committee of the Feinstein Institute for Medical Research, Northwell Health, Manhasset, NY and the Institutional Animal Care and Use Committee at the University of Western Ontario (Protocols 2018-103 and 2018-104).

## Author Contributions

KRL, MAMP, KJT, and VAP designed research. KRL, MEA, HAS, MA-O, AR, YL, PSO, SSC, RG, VFP, and VAP performed research. NMN, YA-A, RG, VFP, and MAMP contributed reagents, analytic tools and knockout and transgenic mice. KRL, HAS, RG, YA-A, CNM, VFP, MAMP, KJT, and VAP analyzed and interpreted data. KRL, KJT, and VAP wrote the manuscript. NMN, CNM, PSO, YA-A, and MAMP provided additional comments to finalize the paper.

### Conflict of Interest Statement

VAP, SSC, and KJT are inventors on patents broadly related to the topic of this paper and have assigned their rights to the Feinstein Institute for Medical Research. YL was employed by SetPoint Medical Corporation (Valencia, CA 91355, USA). The remaining authors declare that the research was conducted in the absence of any commercial or financial relationships that could be construed as a potential conflict of interest.
