# Supplementary figures and images for: Forebrain Cholinergic Signaling Regulates Innate Immune Responses and Inflammation
Source: Front Immunol. 2019 Apr 2;10:585. doi: 10.3389/fimmu.2019.00585 (PMC6455130; doi:10.3389/fimmu.2019.00585)

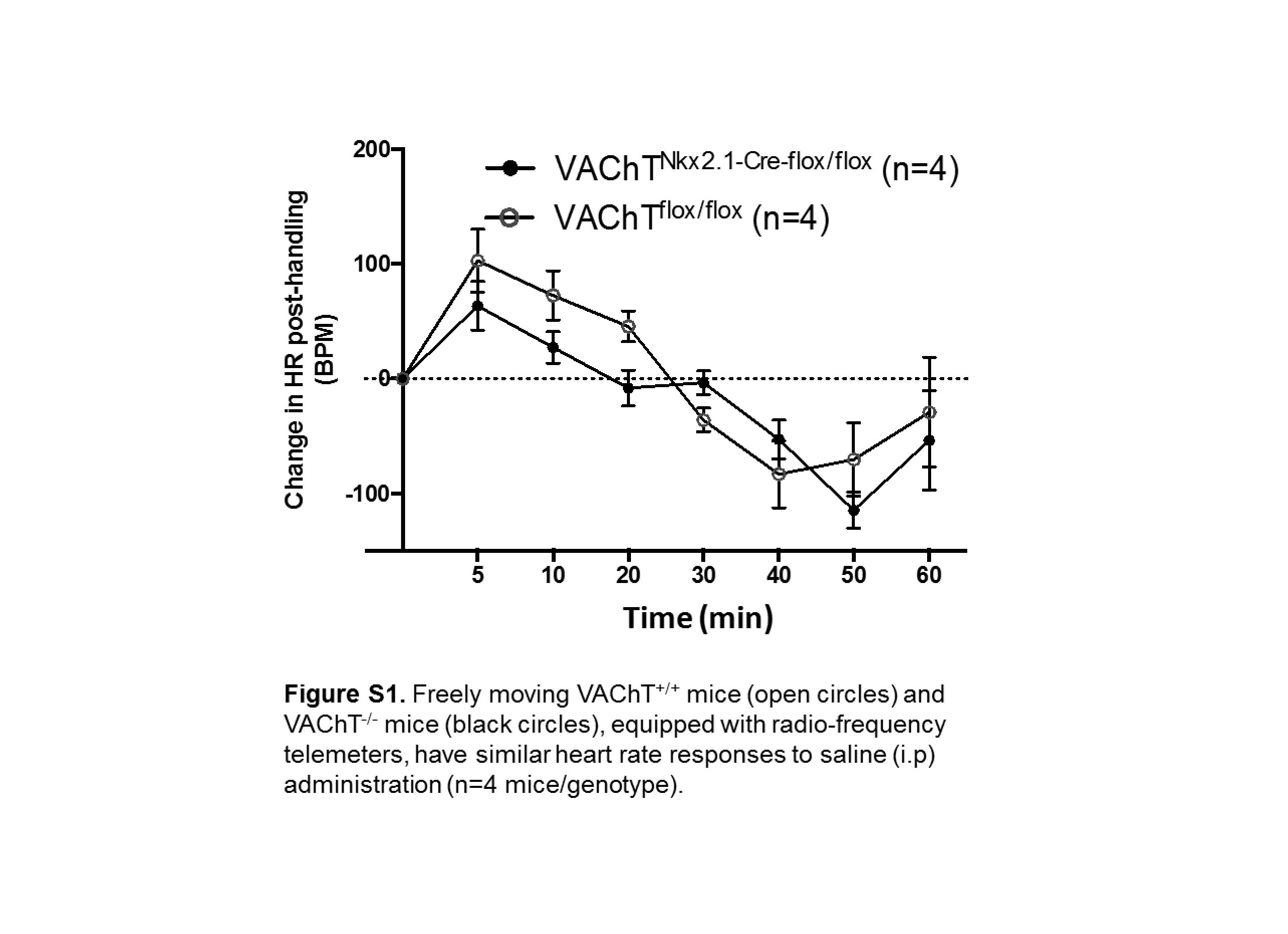

Supplement: Supplementary file 1 [file Image_1.jpg]

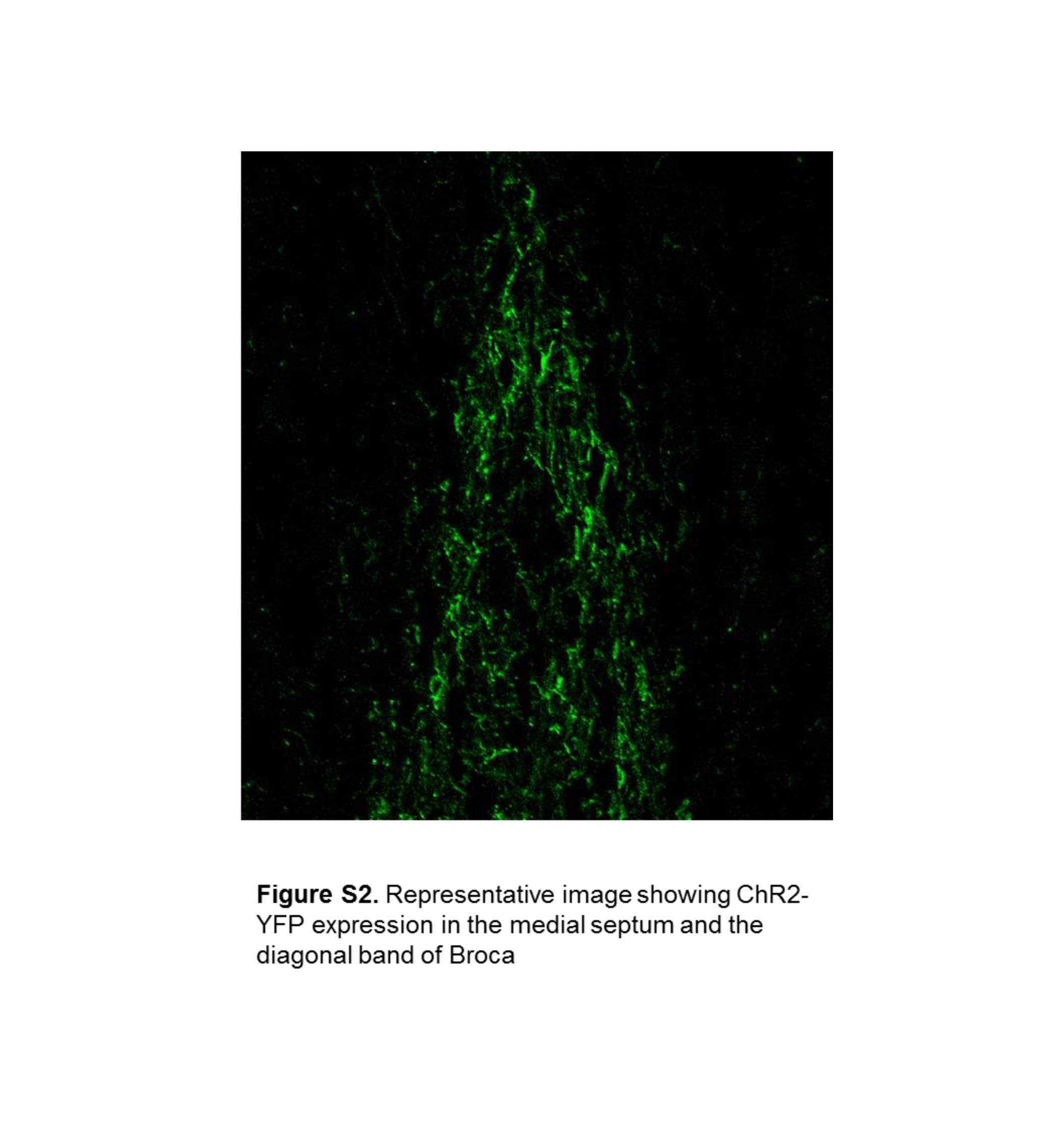

Supplement: Supplementary file 2 [file Image_2.jpg]

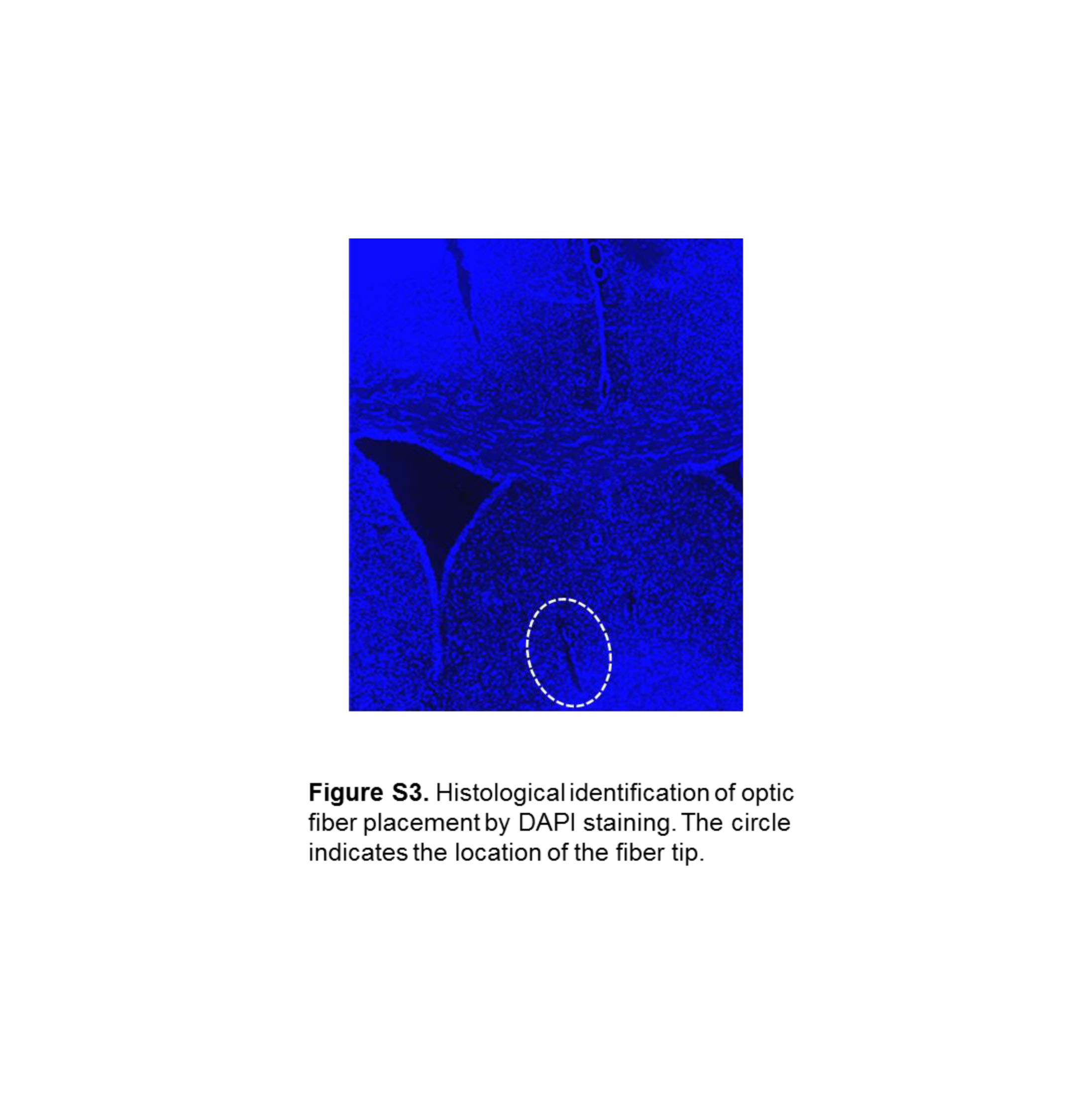

Supplement: Supplementary file 3 [file Image_3.jpg]
