# Supplementary material for: Forebrain Cholinergic Signaling Regulates Innate Immune Responses and Inflammation
Source: Front Immunol. 2019 Apr 2;10:585. doi: 10.3389/fimmu.2019.00585 (PMC6455130; doi:10.3389/fimmu.2019.00585)
Supplement: Supplementary file 4 [file Presentation_1.pdf]

Supplementary Methods

To visualize the correct placement of the optic fiber the following procedure was performed. Mice underwent intracardiac perfusion with 1x PBS followed by 4% paraformaldehyde. Brains were harvested and cryoprotected with subsequent incubations of 15% and 30% sucrose. They were then stored in O.C.T. at -20 ^{0}C. 40 μm sections were collected using a cryostat (Leica Microsystems). Slides were mounted with DAPI-Fluoromount G (Southern Biotech) to visualize the track of the optic fiber with a Zeiss LSM 510 Meta confocal system.
